# Synthetic Porcelain

**Published:** 1916-09

**Authors:** Perry C. Baird

**Affiliations:** Dallas, Texas


### SYNTHETIC PORCELAIN.

BY PERRY C. BAIRD, D. D. S., DALLAS, TEXAS.

In synthetic porcelain we have a filling material which you can vary the manner of working but little to get good results. When you find the best manner of mixing, the proper consistency, the best way of introducing and finishing the filling to obtain best results and can go through these steps in the same manner for each cavity that presents itself for filling, I believe you will always have uniform and beautiful results.

My chief trouble in using the material is in not using it often or continuously enough. I may insert ten or fifteen synthetic fillings one week and the next week two or three or even none. On account of insufficient practice in using this material I attribute my failures. During the weeks in which I do make many fillings is the time I make the most lasting and pleasing fillings. It is not a difficult material to work; on the contrary, it is easy if you comply with a few simple requirements. You can not ignore the directions. The makers of this material have made test after test in order to find the best manner of working this substance, and you may be sure there is a reason for each requirement. It is reasonable for me to see that it takes no more time and pains to insert the best synthetic filling than it does to insert an inferior one.

I no longer use gold in the anterior teeth if a synthetic filling can be used and be not subject to the stress of the opposing teeth.

I tell my patients that we have not yet proven synthetic to be as durable as gold, but that I much prefer it in my own teeth, though I may not think it as lasting. I believe, also, that a cavity filled with synthetic will resist recurrence of decay longer than will one filled with gold.

As for aesthetics and harmony in appearance, there is nothing that equals synthetic porcelain as a filling material. Compare mouths of five or six fillings in the anterior teeth, the cavities of one filled with synthetic and the other with gold. The one filled with gold would be offensive while the one with synthetic would be pleasing.

The first requisite in the manipulation of synthetic is cleanliness. The slab, the spatula and instruments must be absolutely clean, free from former mixes or taint of medicines, grease or other substances.

The spatula must be of agate, neither bone or ivory should be used because particles wear off in the mix which will deteriorate the value of the filling.

The liquid should not be allowed to accumulate and crystallize in the necks of the bottle. The bottle should be kept tightly stopped at all times because the element of the water in the liquid will evaporate and slow setting will result. When the powder and liquid are on the slab the mixing should proceed at once.

The directions say that the average time required for a proper mix is a minute and a quarter and that you may vary from this not more than a quarter of a minute. If the mix is continued too long crystallization will begin before the mass is placed in the cavity, with unhappy results.

The temperature of the slab should be around 70. The slab should not be chilled to a point where moisture condenses. You may reduce the temperature by placing slab and spatula under running water and then wiping dry. The reason for reducing the temperature of the slab is because when the temperature is low more powder may be incorporated with the liquid, making a denser, stronger mass.

The tooth and filling should be isolated from all moisture while the filling is being placed and until it has set. A celluloid strip should be used to cause pressure against the filling until it has set. Under no conditions should the mass be agitated or worked with after it has begun to crystallize because it will crumble and the filling will fail.

After the mass has been placed in the cavity and allowed to remain under the strip with pressure for three minutes, the strip should be removed and the filling immediately coated with cocoa butter to exclude evaporation of moisture and to prevent moisture from reaching it. You should then wait fifteen minutes with the dam still in position. The filling may then be finished, after which it is coated with the varnish and patient dismissed, or after the fifteen minute interval it may be coated with the varnish and the filling finished at a later sitting.

In the preparations of cavities be sure all stained dentin is removed or covered with some opaque cement to prevent the dark shadow showing through the synthetic.

Give all cavities retentive form with square margins. It would be wise to coat the finishing strips and disks with cocoa butter, always revolving disks toward the filling and never toward the margin.

In making your shade select a single color if possible. In the yellow colors use No. 1 to lighten and No. 5 to darken. In the grays use No. 6 to lighten and No. 10 to darken.

It is possible to get such results in color with this material that it almost takes a microscope to distinguish the filling from the tooth. It will fulfill your happiest dreams as to aesthetics and I believe this result may be obtained any time if we have sufficient practice.

I also believe a synthetic failure is due to faulty manipulation. Follow directions and you will have beautiful results.—Texas *Dental Journal.*

